# Do Improvements in Balance Relate to Improvements in Long-Distance Walking Function after Stroke?

**DOI:** 10.1155/2014/646230

**Published:** 2014-07-10

**Authors:** Louis N. Awad, Darcy S. Reisman, Stuart A. Binder-Macleod

**Affiliations:** ^1^Department of Physical Therapy, University of Delaware, 540 South College Avenue, Newark, DE 19713, USA; ^2^Graduate Program in Biomechanics and Movement Science, Newark, DE 19713, USA; ^3^Delaware Clinical and Translational Research Accel Program, Newark, DE 19713, USA

## Abstract

Stroke survivors identify a reduced capacity to walk farther distances as a factor limiting their engagement at home and in community. Previous observational studies have shown that measures of balance ability and balance self-efficacy are strong predictors of long-distance walking function after stroke. Consequently, recommendations to target balance during rehabilitation have been put forth. The purpose of this study was to determine if the changes in balance and long-distance walking function observed following a 12-week poststroke walking rehabilitation program were related. For thirty-one subjects with hemiparesis after stroke, this investigation explored the cross-sectional (i.e., before training) and longitudinal (i.e., changes due to intervention) relationships between measures of standing balance, walking balance, and balance self-efficacy versus long-distance walking function as measured via the 6-minute walk test (6MWT). A regression model containing all three balance variables accounted for 60.8% of the variance in 6MWT performance (_adj_ *R* ^2^ = .584; *F*(3,27) = 13.931; *P* < .001); however, only dynamic balance (FGA) was an independent predictor (*β* = .502) of 6MWT distance. Interestingly, changes in balance were unrelated to changes in the distance walked (each correlation coefficient <.17, *P* > .05). For persons after stroke similar to those studied, improving balance may not be sufficient to improve long-distance walking function.

## 1. Introduction

The recovery of walking function is an ultimate goal of rehabilitation after stroke [1]. Indeed, for a majority of stroke survivors, impairments in their ability to walk farther limit their participation at home and in the community [2]. Unfortunately, current therapies are generally unable to improve the majority of subjects' capacity for community ambulation [3]. Moreover, walking deficits that contribute to reduced endurance and speed persist following rehabilitation [4–6]. A better understanding of the changes underlying improvements in long-distance walking function following walking rehabilitation would inform future efforts and define specific targets for gait intervention.

Recent observational studies have shown balance to be a powerful predictor of poststroke walking function [7–13] and a variable related to quality of life after stroke [14]. Specifically, individuals with better balance abilities typically present with better walking function. Based on such findings, recommendations to target balance during poststroke rehabilitation are commonly put forth. However, basing interventions on the results of cross-sectional studies may not be appropriate as cross-sectional analyses only measure the relationship between variables at a single time point. That is, a strong cross-sectional relationship between walking function and balance does not provide evidence for the potential effect on walking function of gait intervention that improves balance. Thus, this study aimed to determine if the changes in balance and long-distance walking function observed following a 12-week poststroke walking rehabilitation program were related and if these findings were consistent with prior work carried out on slower [9] and faster [13] walkers in the chronic phase of stroke recovery.

## 2. Methods

### 2.1. Subjects

Thirty-one subjects with hemiparesis after stroke were studied. These subjects were those randomized to the treatment arms (see Section 2.2) of a clinical trial at the University of Delaware (Table 1). Subjects were those with single cortical or subcortical stroke, in the chronic phase of recovery (>six months after stroke), who had observable gait deficits but could ambulate independently for six minutes without orthotic support, were able to follow instruction and communicate with the investigators, and were able to passively dorsiflex the ankle to a neutral position with the knee extended and passively extend the hip at least ten degrees. A history of multiple strokes, cerebellar stroke, lower extremity joint replacement, orthopedic conditions that limited walking ability, neglect and hemianopia, unexplained dizziness in the last 6 months, and chest pain or shortness of breath without exertion excluded subjects from participating. All subjects signed written informed consent forms. This study was approved by the University of Delaware's institutional review board.

### 2.2. Intervention

Subjects participated in a treadmill and overground walking retraining program at a frequency of 3 sessions per week for 12 weeks. On the treadmill, subjects walked five bouts of 6 minutes at the maximum walking speed they could maintain for four minutes. Subjects either walked with or without functional electrical stimulation applied to the paretic ankle plantarflexors during late stance and dorsiflexors during swing phase in an alternating pattern of one minute on to one minute off. The FES component of the intervention has previously been described [15]. Subjects were connected to an overhead harness system for safety while on the treadmill, but no body weight was supported via the harness. Five-minute rest breaks were provided between each of the walking bouts. A total of 36 minutes of walking practice were planned for each session with the final 6 minutes of walking occurring over ground. This bout of overground walking was included to promote carryover of gains made on the treadmill and focused on independent walking, dynamic balance, and endurance as subjects walked at their fastest pace, performing regular turns and terrain transitions.

### 2.3. Outcomes

The 6-minute walk test (6MWT) [12, 16] served as this study's a priori measure of long-distance walking function. Balance abilities were measured via the Berg Balance Scale (BERG) [17], the functional gait assessment (FGA) [18], and the Activities-Specific Balance Confidence (ABC) Scale [19]. As such, balance impairments in the standing (BERG), walking (FGA), and self-efficacy (ABC) constructs were quantified. Change scores for each variable were determined as the difference between posttraining and pretraining performance. These measures have been shown to be reliable and valid in individuals after stroke [17–19]. Subjects were allowed to use their regular assistive devices during testing if necessary. All testing was conducted by a licensed physical therapist.

### 2.4. Data Analyses

Statistical analyses were performed using the SPSS 21 and G Power 3.1 software packages. The threshold for significance was set to *P* < 0.05. The Shapiro-Wilk test determined data normality. The paired *t*-test or the Wilcoxon signed rank test—pending data normality—was used to determine whether changes in each variable followed the intervention period. Individual subject changes were compared to known minimal detectable change (MDC) values. Pearson *r* or Spearman rho correlation analyses measured the cross-sectional and change-score relationships between each balance measure versus the 6MWT. Multiple regression was used to quantify the independent contribution to performance in the 6MWT or to changes in the 6MWT of the balance measures found to be significantly related in the correlation analyses.

## 3. Results

Complete data sets were available for all subjects (*N* = 31). An a priori power analysis revealed that with 31 subjects, at an alpha of 0.05, this investigation would be powered at a level of 80% to detect a significant *r* = 0.453 (*R* ^2^ of 0.21).

**Figure 1:**
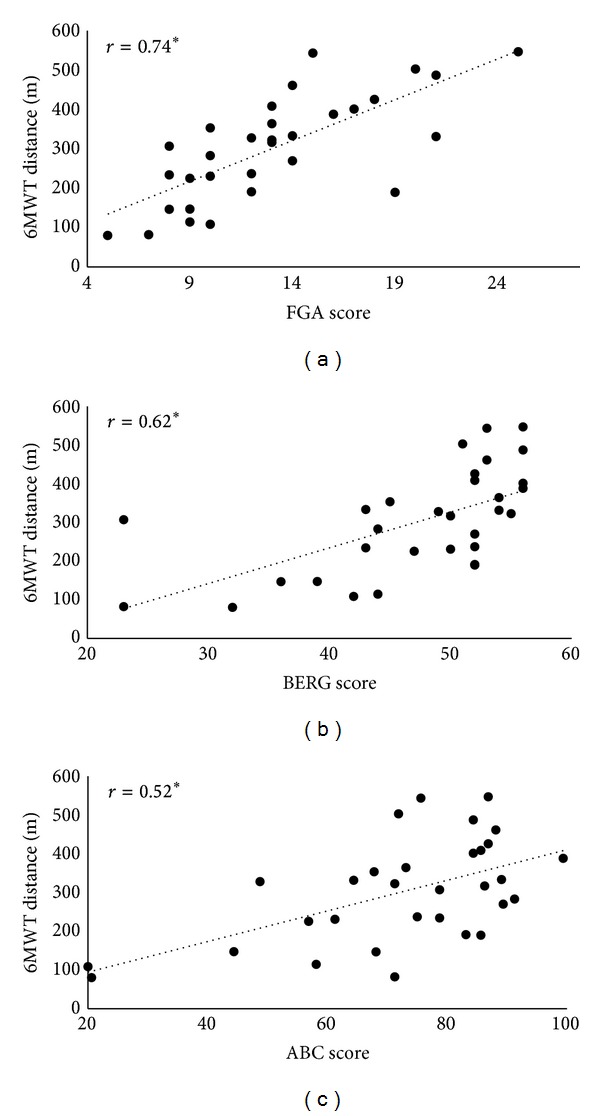
Scatter plots presenting the relationships before training between long-distance walking function (6MWT) versus walking balance (functional gait assessment (FGA), panel (a)), standing balance (Berg Balance Scale (BERG), panel (b)), and balance self-efficacy (Activities Specific Balance Confidence (ABC) Scale, panel (c)). Each of these variables was strongly related to long-distance walking function before training. ∗ = *P* ≤ 0.05.

Consistent with previous work, correlation analyses demonstrated that each balance construct strongly correlated with long-distance walking function (correlation coefficients ranged from 0.517 to 0.736, *P* < 0.05; see Figure 1). Multiple regression analysis revealed that a model containing all three balance measures accounted for 60.8% of the variance in 6MWT performance (_adj_ *R* ^2^ = 0.584; *F*(3,27) = 13.931; *P* < 0.001); however, only dynamic balance (FGA) independently predicted the distance walked during the 6MWT (*β* = 0.502; *t* = 2.76; *P* = 0.01). It should be noted that balance self-efficacy (ABC) trended towards significance as an independent predictor (*β* = 0.257; *t* = 1.859; *P* = 0.074), but interestingly standing balance (BERG) did not (*β* = 0.152; *t* = 0.871; *P* = 0.391).

**Figure 2:**
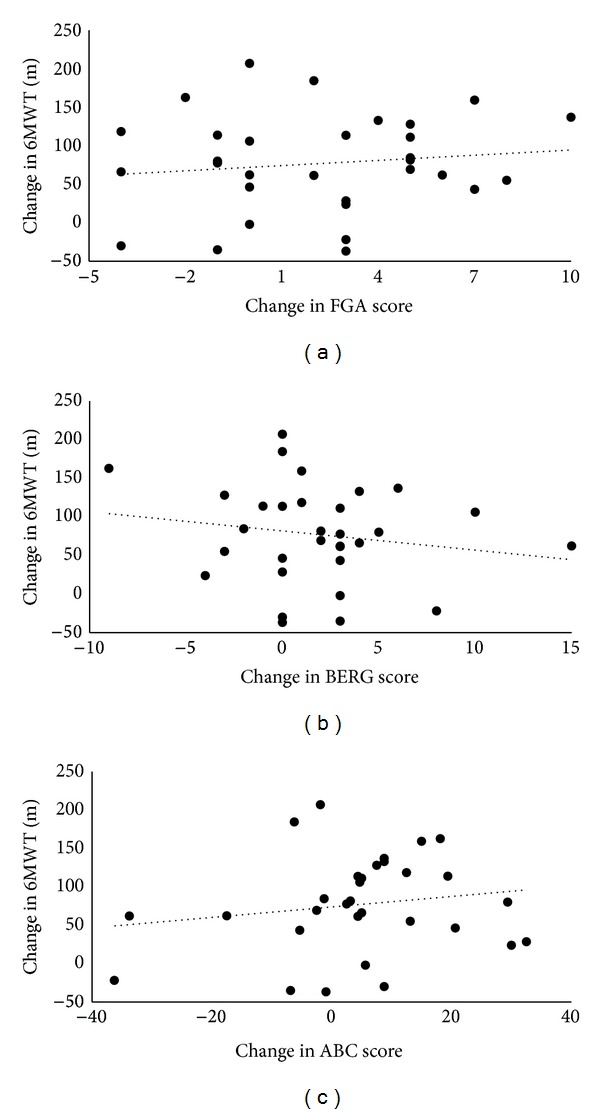
Scatter plots presenting the relationships between changes in long-distance walking function (6MWT) versus changes in walking balance (functional gait assessment (FGA), panel (a)), standing balance (Berg Balance Scale (BERG), panel (b)), and balance self-efficacy (Activities Specific Balance Confidence (ABC) Scale, panel (c)). Even though each balance variable was strongly related to 6MWT performance at baseline (Figure 1), no significant change relationships were detected.

Improvements in long-distance walking function, standing balance, walking balance, and balance self-efficacy were observed following the 12-week intervention period (see Table 2). Ten subjects changed their FGA score by more than 5 points, which is the MDC previously established for the FGA in persons with stroke [20]. Nine subjects changed their BERG score by more than 4 points, which is the MDC previously established for the BERG in those with chronic stroke [21]. Eleven subjects changed their ABC score more than the previously established MDC of 13.8% [22]. Despite these statistically significant improvements in each balance measure after versus before training (see Table 2), changes in balance did not correlate with changes in long-distance walking function (each correlation coefficient < 0.17, *P* > 0.05; see Figure 2). Even when considering just those subjects whose balance changes exceeded known MDC values, changes in each variable did not relate to changes in long-distance walking function (each correlation coefficient < 0.31, *P* > 0.05).

## 4. Discussion

The development of effective targeted walking interventions depends on the valid identification of deficits that, when improved, will result in the recovery of walking function. The findings of this study suggest that basing the development of targeted interventions solely on the findings of cross-sectional analyses may not be appropriate. Indeed, despite being highly predictive of walking function before training, changes in balance did not relate to the changes in walking function observed after training. Moreover, to our knowledge, this is the first study to demonstrate that standing balance does not independently contribute to walking function if dynamic balance and balance self-efficacy are controlled.

Consistent with the findings of the present investigation, Bowden and colleagues reported that changes in standing balance (BERG), balance confidence (ABC), and walking balance (FGA in our study, the Dynamic Gait Index in theirs) did not correlate with changes in self-selected walking speed (SSWS) [23]. Bowden et al. considered a similar stroke population (age: 58.74 ± 12.97 y; 1.89 ± 1.37 y after stroke) which, although walked at a slower pace than our cohort (average SSWS: 0.48 ± 0.19 m/s), would still be classified functionally as a population of limited community ambulators [24]. Taken together, the findings of Bowden et al. and the present study may carry significant implications. Indeed, two different intervention studies measured marked improvements in two different measures of walking function that were not related to improvements in standing balance, walking balance, or balance self-efficacy. Such findings suggest that, for individuals similar to those studied, deficits in balance may not be the primary limiting factors of short- and long-distance walking function after a stroke. That is, improving balance may be insufficient to improve walking function after stroke.

Previous findings by Patterson and colleagues [9] and Schmid and colleagues [13] offer an interesting framework to consider the present findings. First, Patterson et al. demonstrated that, for subjects walking slower than 0.48 m/s, impairments in standing balance explained the largest portion of 6MWT variability [9]. Second, Schmid et al. demonstrated that, for subjects walking as fast as 1.33 m/s, balance self-efficacy independently contributed to activity and participation after stroke [13]. Our finding that changes in balance do not relate to changes in walking function may be explained by the fact that the majority of subjects presently studied walked at speeds between those studied by Patterson and Schmid. Interestingly, Patterson et al. also demonstrate that, for those walking faster than 0.48 m/s, variability in cardiovascular fitness—and not balance—explained 6MWT variability [9]. Taken together, these findings suggest that some aspect of balance—capacity or confidence—may be a key limiter of walking function for the slowest and fastest walkers after stroke; however, for those walking at relatively moderate speeds, other factors may be contributing to reduced walking performance. The elucidation of these factors in future studies is critical to the development of targeted walking interventions for limited community ambulators in the chronic phase of stroke recovery.

Patient-specific characteristics should be considered when planning interventions. For those patients with a high fall risk or history of falls, balance may be the primary limiting factor to improved walking function and should thus be a target during rehabilitation. Indeed, for these individuals, walking may be optimized to prevent falls. For example, Blennerhassett and colleagues demonstrate that community-dwelling individuals after stroke were more likely to fall and walk less distance during the 6MWT if they were concerned about falling, had limited confidence, and performed worse on measures of dynamic balance [25]. As such, a thorough subjective report should be considered in conjunction with objective measures of physical function when deciding on the best interventions for patients.

### 4.1. Study Limitations

This study investigated the relationships between changes in balance and changes in walking function following activity-based interventions that did not directly target balance abilities. For subjects whose balance deficits are limiting walking function, specific balance training may be necessary to produce changes in balance sufficient to alter walking function. However, it should be noted that both the treadmill and overground portions of the interventions studied did indirectly challenge balance abilities and a number of subjects had meaningful improvements (larger than known MDCs) in each balance measure considered. Even when evaluating the relationships between improvements in walking function versus improvements in balance for just these subjects, the relationships were weak and not significant.

### 4.2. Conclusions

For limited community ambulators in the chronic phase of stroke recovery, improving balance may be insufficient to improve long-distance walking function. Moreover, basing interventions on cross-sectional relationships may not be appropriate.

## Figures and Tables

**Table 1:** Subject characteristics.

Subject	Sex	Age, y	Time since stroke, y	Side of hemiparesis	Walking speed, m/s
1	Male	63.5	7.99	Right	0.92
2	Female	63.2	30.52	Right	0.94
3	Female	65.4	22.90	Left	0.20
4	Female	64.9	24.65	Right	0.70
5	Male	60.0	2.67	Left	0.41
6	Female	47.6	3.77	Left	0.74
7	Male	54.2	8.55	Left	1.16
8	Female	55.5	1.87	Left	0.80
9	Male	57.8	0.54	Right	0.59
10	Male	60.1	0.93	Right	1.06
11	Male	68.7	2.86	Left	0.79
12	Male	49.0	1.59	Right	0.97
13	Female	55.1	0.90	Right	0.45
14	Female	63.0	1.19	Right	0.27
15	Male	42.7	0.57	Left	0.61
16	Male	45.1	3.35	Left	0.24
17	Male	57.5	0.59	Left	0.87
18	Male	67.9	0.77	Left	0.65
19	Female	56.7	0.94	Left	0.12
20	Male	70.7	1.71	Left	0.84
21	Female	48.7	7.94	Right	0.60
22	Male	54.9	1.66	Left	0.74
23	Male	69.5	8.25	Right	0.72
24	Male	55.1	5.23	Left	0.88
25	Male	55.7	0.73	Left	0.33
26	Male	61.5	6.94	Right	1.01
27	Male	71.3	0.55	Left	0.88
28	Female	56.0	3.51	Left	1.18
29	Male	25.3	1.70	Left	1.51
30	Male	43.2	7.06	Left	1.02
31	Female	64.2	1.56	Left	0.93
	Male: 65%^a^	57.2 (9.9)^b^	1.87 (0.94–7.00)^c^	Right: 36%^a^	0.75 (0.32)^b^

^a^Percent; ^b^mean (SD);^ c^median (IQR).

**Table 2:** Pretraining and change-score values.

	Measures of central tendency and precision		*T *or* Z *statistic	*P* value
	Pretraining	Change		
	6MWT (distance)			
Mean (SD)	301 (134)	77 (63)	−6.779	.000
95% CI	252–351	54–100		

	BERG (score)			
Mean (SD)	47.29 (8.92)	1.83 (4.36)	−2.348	.013
95% CI	43–52	0.24–3.44		

	FGA (score)			
Mean (SD)	13.06 (4.75)	2.20 (3.65)	−3.350	.001
95% CI	11.32–14.81	0.86–3.53		

	ABC (score)			
Median (IQR)	76 (22)^a^	4 (15)^a^	−2.205^b^	.014
95% CI	65–79	−0.93–10.39		

6MWT: six-minute walk test; BERG: Berg Balance Scale; FGA: functional gait assessment; ABC: Activities Specific Balance Confidence Scale.

If data were not normally distributed, median (IQR)^a^ and *Z* score^b^ are presented.

## References

[1] Bohannon RW, Horton MG, Wikholm JB (1991). Importance of four variables of walking to patients with stroke. *International Journal of Rehabilitation Research*.

[2] Combs SA, Van Puymbroeck M, Altenburger PA, Miller KK, Dierks TA, Schmid AA (2013). Is walking faster or walking farther more important to persons with chronic stroke?. *Disability and Rehabilitation*.

[3] Dickstein R (2008). Rehabilitation of gait speed after stroke: a critical review of intervention approaches. *Neurorehabilitation and Neural Repair*.

[4] Mayo NE, Wood-Dauphinee S, Ahmed S (1999). Disablement following stroke. *Disability and Rehabilitation*.

[5] Olney SJ, Richards C (1996). Hemiparetic gait following stroke. Part I: characteristics. *Gait & Posture*.

[6] Richards CL, Malouin F, Dean C (1999). Gait in stroke: assessment and rehabilitation. *Clinics in Geriatric Medicine*.

[7] Ng SSM (2011). Contribution of subjective balance confidence on functional mobility in subjects with chronic stroke. *Disability and Rehabilitation*.

[8] Ng SSM (2010). Balance ability, not muscle strength and exercise endurance, determines the performance of hemiparetic subjects on the timed-sit-to-stand test. *American Journal of Physical Medicine & Rehabilitation*.

[9] Patterson SL, Forrester LW, Rodgers MM (2007). Determinants of walking function after stroke: differences by deficit severity. *Archives of Physical Medicine and Rehabilitation*.

[10] Carvalho C, Willén C, Sunnerhagen KS (2008). Relationship between walking function and one-legged bicycling test in subjects in the later stage post-stroke. *Journal of Rehabilitation Medicine*.

[11] Eng JJ, Chu KS, Dawson AS, Kim CM, Hepburn KE (2002). Functional walk tests in individuals with stroke: relation to perceived exertion and myocardial exertion. *Stroke*.

[12] Pohl PS, Duncan PW, Perera S (2002). Influence of stroke-related impairments on performance in 6-minute walk test. *Journal of Rehabilitation Research and Development*.

[13] Schmid AA, van Puymbroeck M, Altenburger PA (2012). Balance and balance self-efficacy are associated with activity and participation after stroke: a cross-sectional study in people with chronic stroke. *Archives of Physical Medicine and Rehabilitation*.

[14] Schmid AA, van Puymbroeck M, Altenburger PA, Miller KK, Combs SA, Page SJ (2013). Balance is associated with quality of life in chronic stroke. *Topics in Stroke Rehabilitation*.

[15] Awad LN, Reisman DS, Kesar TM, Binder-Macleod SA (2014). Targeting paretic propulsion to improve post-stroke walking function: a preliminary study. *Archives of Physical Medicine and Rehabilitation*.

[16] Fulk GD, Echternach JL, Nof L, O'Sullivan S (2008). Clinometric properties of the six-minute walk test in individuals undergoing rehabilitation poststroke. *Physiotherapy Theory and Practice*.

[17] Berg K, Wood-Dauphinee S, Williams JI (1995). The balance scale: reliability assessment with elderly residents and patients with an acute stroke. *Scandinavian Journal of Rehabilitation Medicine*.

[18] Wrisley DM, Marchetti GF, Kuharsky DK, Whitney SL (2004). Reliability, internal consistency, and validity of data obtained with the functional gait assessment. *Physical Therapy*.

[19] Powell LE, Myers AM (1995). The Activities-specific Balance Confidence (ABC) Scale. *The Journals of Gerontology A: Biological Sciences and Medical Sciences*.

[20] Lin J-H, Hsu M-J, Hsu H-W, Wu H-C, Hsieh C-L (2010). Psychometric comparisons of 3 functional ambulation measures for patients with stroke. *Stroke*.

[21] Flansbjer U-B, Blom J, Brogårdh C (2012). The reproducibility of Berg Balance Scale and the Single-leg Stance in chronic stroke and the relationship between the two tests. *PM & R*.

[22] Botner EM, Miller WC, Eng JJ (2005). Measurement properties of the activitites-specific balance confidence scale among individuals with stroke. *Disability and Rehabilitation*.

[23] Bowden MG, Behrman AL, Neptune RR, Gregory CM, Kautz SA (2013). Locomotor rehabilitation of individuals with chronic stroke: difference between responders and nonresponders. *Archives of Physical Medicine and Rehabilitation*.

[24] Perry J, Garrett M, Gronley JK, Mulroy SJ (1995). Classification of walking handicap in the stroke population. *Stroke*.

[25] Blennerhassett JM, Dite W, Ramage ER, Richmond ME (2012). Changes in balance and walking from stroke rehabilitation to the community: a follow-up observational study. *Archives of Physical Medicine and Rehabilitation*.

